# Supplementary material for: Streptococcus salivarius as an Important Factor in Dental Biofilm Homeostasis: Influence on Streptococcus mutans and Aggregatibacter actinomycetemcomitans in Mixed Biofilm
Source: Int J Mol Sci. 2023 Apr 14;24(8):7249. doi: 10.3390/ijms24087249 (PMC10139097; doi:10.3390/ijms24087249)
Supplement: Supplementary file 1 [file ijms-24-07249-s001.zip › ijms-2311254-supplementary.pdf]

Streptococcus salivarius as important factor in dental biofilm homeostasis: influence on *Streptococcus mutans* and *Aggregatibacter actinomycetemcomitans* in mixed biofilm

Gabrijela Begić [1], Ivana Jelovica Badovinac[2], Ljerka Karleuša[3], Kristina Kralik[4], Olga Cvijanovic Peloza[5], Davor Kuiš[6,7,8,*] and Ivana Gobin[1]

Table S1. Comparison of the number (log_{10}CFU/mL) of individual bacterial species in non-salivarius and salivarius mixed biofilm

	**d-PTFE-Permamem**			**d-PTFE-Cytoplast**			**Dentin**			**Hydroxyapatite**		
	non-salivarius biofilm	salivarius biofilm	*P**	non-salivarius biofilm	salivarius biofilm	*P**	non-salivarius biofilm	salivarius biofilm	*P**	non-salivarius biofilm	salivarius biofilm	*P**
	Median (95% CI for median)			Median (95% CI for median)			Median (95% CI for median)			Median (95% CI for median)		
**0 h**												
S. salivarius	-	4,716 (4,51 – 4,83)	-	-	6,312 (6,26 – 6,36)	-	-	6,989 (6,21 – 7,16)	-	-	6,317 (6,28 – 6,36)	-
S. mutans	6,597 (6,55 – 6,39)	6,491 (6,24-6,47)	**0,002**	6,311 (6,26 – 6,36)	6,137 (6,05 – 6,25)	**0,005**	6,782 (6,76 – 6,79)	6,331 (6,48 - 6,59)	**0,004**	6,618 (6,58 – 6,64)	1 (1 – 1)	**0,002**
S. oralis	5,597 (5,55 – 5,64)	5,439 (5,39 – 5,49)	**0,002**	5,190 (5,09 – 5,25)	4,977 (4,18 – 5,13)	**0,01**	5,597 (5,56 – 5,65)	7,064 (6,96 – 7,11)	**0,004**	6,296 (6,27 - 6,36)	6,199 (6,15 – 6,24)	**0,002**
A.actinomycete mcomitans	-	-	-	-	-	-	-	-	-	-	-	-
**24 h**												
S. salivarius	-	4,484 (4,42 – 4,53)	-	-	6,505 (6,45 – 6,55)	-	-	7,169 (4,13 – 7,20)	-	-	7,250 (7,22 – 7,28)	-
S. mutans	6,597 (6,56 – 6,63)	5,312 (5,25-5,38)	**0,002**	7,055 (7,0 – 7,09)	6,021 (5,91-6,11)	**0,002**	7,306 (7,26-7,36)	5,360 (5,26 - 5,48)	**0,002**	7,306 (7,24 – 7,33)	2,301 (2,0 – 2,58)	**0,004**
S. oralis	6,648 (6,61 – 6,67)	5,591 (5,52 – 5,71)	**0,002**	7,085 (7,07 – 7,09)	6,021 (5,98 – 6,08)	**0,004**	7,484 (7,44 – 7,50)	6,703 (6,66 – 6,74)	**0,002**	6,771 (6,74 – 6,82)	7,161 (7,09 – 7,24)	**0,002**

A.actinomycete mcomitans	7,916 (7,85 – 7,99)	5,079 (4,94 – 5,07)	**0,002**	7,708 (6,88 – 7,78)	6,959 (6,10 – 6,99)	**0,04**	5,597 (5,55 – 5,64)	5,823 (5,78 – 5,86)	**0,002**	7,169 (7,15 – 7,19)	6,511 (6,45 – 6,62)	**0,002**
**72 h**												
S. salivarius	-	7,491 (7,28 – 7,59)	**-**	-	6,397 (6,28 – 6,48)	**-**	-	5,968 (5,88 – 6,03)	**-**	-	7,060 (7,0 – 7,20)	**-**
S. mutans	6,703 (6,69 – 6,72)	7,105 (6,96-7,14)	**0,004**	5,539 (5,03-5,76)	6,100 (5,96-6,24)	**0,002**	6,892 (6,88 – 6,90)	5,484 (5,41 – 5,61)	**0,004**	7,289 (7,19 – 7,32)	5,332 (5,26 – 5,41)	**0,002**
S. oralis	6,069 (4,91 – 6,14)	7,039 (6,93 – 7,14)	**0,002**	6,190 (6,15 – 6,27)	6,484 (6,39 – 6,55)	**0,004**	6,454 (6,09 – 6,49)	5,167 (4,92 – 5,46)	**0,002**	5,266 (5,15 – 5,46)	6,021 (2,93 – 6,11)	**0,002**
A.actinomycete mcomitans	7,039 (6,93 – 7,16)	6,423 (6,28 – 6,49)	**0,002**	7,175 (7,09 – 7,20)	6,707 (6,64 – 6,79)	**0,002**	8,176 (8,15 – 8,28)	6,379 (6,28 –6,48)	**0,004**	7,839 (7,80 – 7,88)	6,719 (6,67 – 6,79)	**0,002**

95% CI – 95% Confidence interval: *Mann Whitney U test; Bold denotes significant
